# Bone Mineral Densities and Mechanical Properties of Retrieved Femoral Bone Samples in relation to Bone Mineral Densities Measured in the Respective Patients

**DOI:** 10.1100/2012/242403

**Published:** 2012-11-27

**Authors:** Yvonne Haba, Ralf Skripitz, Tobias Lindner, Martin Köckerling, Andreas Fritsche, Wolfram Mittelmeier, Rainer Bader

**Affiliations:** ^1^Biomechanics and Implant Technology Research Laboratory, Department of Orthopaedics, University Medicine Rostock, 18057 Rostock, Germany; ^2^Inorganic Solid State Chemistry Group, Institute of Chemistry, University of Rostock, 18057 Rostock, Germany

## Abstract

The bone mineral density (BMD) of retrieved cancellous bone samples is compared to the BMD measured *in vivo* in the respective osteoarthritic patients. Furthermore, mechanical properties, in terms of structural modulus (*E*_*s*_) and ultimate compression strength (*σ*_max_) of the bone samples, are correlated to BMD data. Human femoral heads were retrieved from 13 osteoarthritic patients undergoing total hip replacement. Subsequently, the BMD of each bone sample was analysed using dual energy X-ray absorptiometry (DXA) as well as ashing. Furthermore, BMDs of the proximal femur were analysed preoperatively in the respective patients by DXA. BMDs of the femoral neck and head showed a wide variation, from 1016 ± 166 mg/cm^2^ to 1376 ± 404 mg/cm^2^. BMDs of the bone samples measured by DXA and ashing yielded values of 315 ± 199 mg/cm^2^ and 347 ± 113 mg/cm^3^, respectively. *E*_*s*_ and *σ*_max_ amounted to 232 ± 151 N/mm^2^ and 6.4 ± 3.7 N/mm^2^. Significant correlation was found between the DXA and ashing data on the bone samples and the DXA data from the patients at the femoral head (*r* = 0.85 and 0.79, resp.). *E*_*s*_ correlated significantly with BMD in the patients and bone samples as well as the ashing data (*r* = 0.79, *r* = 0.82, and *r* = 0.8, resp.).

## 1. Introduction

Dual energy X-ray absorptiometry (DXA), used to evaluate bone quality and predict the risk of bone fracture [1], represents a functional diagnostic tool to determine the mineral density of human femoral bone samples. Bone quality and fracture risk depend on geometric parameters and properties of the material [2]. In order to clarify the relationship between mechanical properties and bone mineral density (BMD), various experimental studies with different measurement techniques have been carried out [3–6]. Radiographic analyses such as DXA or quantitative-computed tomography (QCT) are standard diagnostic tools for assessment of BMD in patients. Ashing has also been used experimentally for *in vitro* determination of BMD [7–9]. However, a comparison of mechanical properties as well as the BMD of retrieved bone samples measured by ashing and DXA with BMD analysis of the respective patients as measured by DXA has not as yet been performed.

To determine the mechanical properties, for example, the structural modulus (*E*_*s*_) or ultimate compression strength (*σ*_max⁡_), of human cancellous bone, uniaxial compression tests have been used in most cases [4, 9–11]. Ebbesen et al. [12] analysed the relationship between *σ*_max⁡_, DXA, QCT samples, and ashing samples of the lumbar vertebral body *in vitro* using bone samples. Fazzalari et al. [13] also compared BMD and mechanical properties of human osteoarthritic femoral cancellous bone samples *in vitro*.

The aim of the present study is to compare the *in vivo* measured BMD of the femur, that is, femoral head and neck, in osteoarthritic patients with the BMD of cylindrical cancellous bone samples retrieved from the respective patients after undergoing total hip replacement and intraoperative removal of the femoral head. Furthermore, mechanical properties, that is, structural modulus (*E*_*s*_) and ultimate compression strength (*σ*_max⁡_), of the bone samples were compared with their BMD as measured by DXA and ashing as well as the BMD measured *in vivo* in patients by DXA.

## 2. Materials and Methods

### 2.1. Sample Preparation

Human femoral heads were retrieved from 13 female donors aged between 53 and 76 years undergoing primary total hip replacement and stored postoperatively for a maximum of 24 hours at +6 to 8°C. Subsequently, the femoral heads were frozen in small sealed containers at −20°C [14]. The specimens were moved 12 hours before testing to the refrigerator (+6° to +8°C) and were frozen at −20°C between the different tests. The DXA investigations were performed first, followed by analysis of the mechanical properties and finally by ashing the bone samples. All tests were conducted at room temperature. The tests were approved by the local Ethical Committee of the University of Rostock (A 2009 38). 

According to DIN 50106 [15], cylindrical bone samples from the centre of the femoral head with a height to diameter ratio between 1 and 2 were used in order to avoid buckling of the test samples during the compression tests. A cylindrical sample approximately 30 mm in length and 12 mm in diameter was cut from each femoral head using a diamond hollow drill (Günther Diamantwerkzeuge, Idar-Oberstein, Germany). The drill was positioned on the femoral head at the base of the ligamentum capitis femoris aligned with the femoral neck axis. The endplates of the bone cylinders were prepared in a tube-like template [10] to assure parallel alignment of the cancellous bone cylinders and an exact length of 15 mm.

### 2.2. DXA

**Figure 1:**
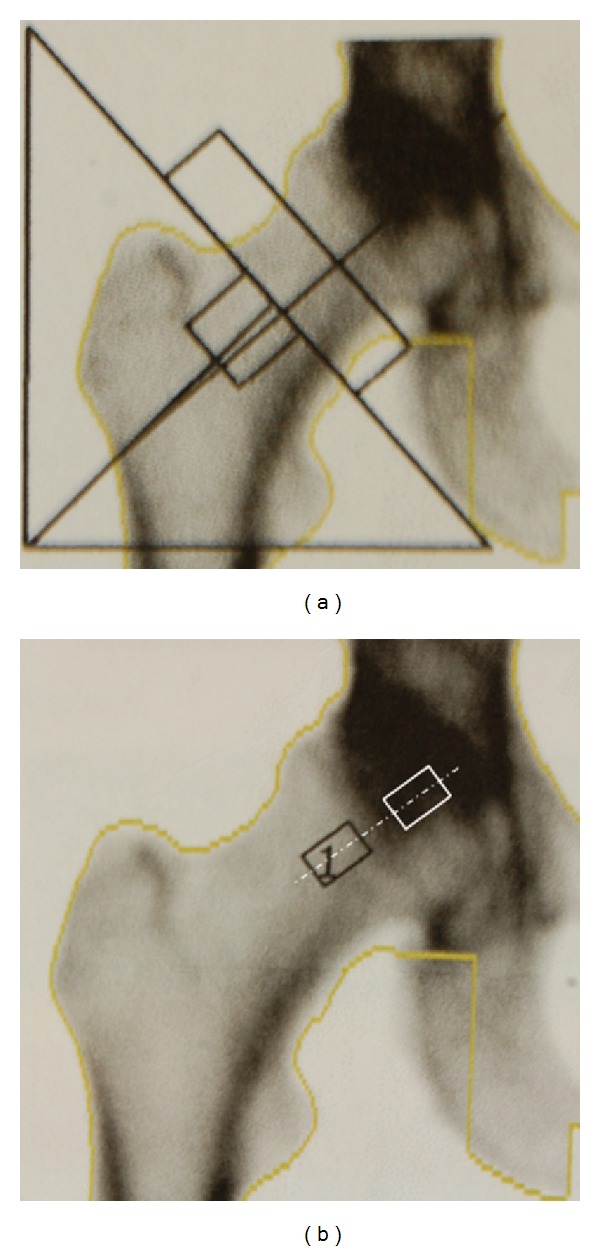
Standard BMD measurement of the patient femoral neck using DXA (a). Bone sample position for *in vivo* measurement of the bone sample (black) and the position of the retrieved cancellous bone sample (white) (b).

BMD analysis of each patient was performed using the standardized region of interest (ROI) of the femoral neck as well as an adaptable ROI of the femoral head referencing the geometry of the cancellous bone samples. Moreover, the BMD measurements on the cylindrical samples were conducted with a DXA device (Lunar Prodigy, General Electric (GE) Healthcare, Munich, Germany) using the research option (electrical potential of 76 kV and electrical current of 0.15 mA). Calibration control of the Lunar Prodigy device was performed daily based on a cuboid calibration phantom (200 × 130 × 60 mm) with three different BMD bone equivalent chambers with values of 0.5 g/cm², 1.0 g/cm², and 1.5 g/cm². The BMD was obtained by using the DXA software enCORE*™* 2007 (version 11.40.004, General Electric (GE) Medical Systems, Madison, WI, USA). The preoperatively performed DXA of the patient was used to set a similar ROI position on the bone samples in the femoral head as shown in Figure 1. In order to avoid the overlap by the acetabulum while performing the DXA measurements, the ROI and real sample positions differed approximately 20 mm in the longitudinal axis. The *T*-score measured in the present study indicates the number of standard deviations above or below the mean for a healthy thirty-year-old normal population with the same gender and ethnicity as the patient. The *T*-score determination is usable in standardised patient measurements, but not applicable for *in vitro* bone samples. According to the criteria of the World Health Organization (WHO), normal *T*-scores range up to −1. Osteopenia is defined as between −1 and −2.5 and osteoporosis as less than −2.5. 

### 2.3. Uniaxial Compression Test

The mechanical properties were derived from a uniaxial compression test assessing the structural modulus (*E*_*s*_) and the ultimate compression strength (*σ*_max⁡_). According to Hooke's law (1), *E*_*s*_ provides a linear relationship between stress (*σ*) and strain (*ε*). Stress and strain data were calculated from the linear part of the load-displacement curve. The ultimate compression strength (*σ*_max⁡_) was reached at the maximum of the compression stress curve during the test. A universal testing machine (Z050, Zwick/Roell, Ulm, Germany) was used to apply axial compression loads to investigate the cylindrical cancellous bone samples. The test speed was set to 5 mm/min [12] until a total displacement of 4 mm was reached:

(1) $$\begin{array}{c} E_{s}=\frac{\sigma }{\varepsilon }. \end{array}$$

### 2.4. Ashing

After compression testing the cylindrical bone samples were combusted in a tube furnace (Nabertherm, Lilienthal, Germany) at 800°C for 5 h [16]. The BMD was calculated as ash weight divided by the gross sample volume [17].

### 2.5. Statistical Analysis

The linear correlation coefficient (*r*) between the different BMDs as well as their mechanical properties was determined by the Pearson correlation. The statistical analysis was determined with the SPSS, Version 20 (SPSS Inc., Chicago, IL, USA) software package. The significance level was set at *P* < 0.05.

## 3. Results

In the present investigation we compared BMDs (DXA and ashing) and mechanical properties (*E*_*s*_ and *σ*_max⁡_) of retrieved femoral bone samples to the preoperatively measured BMDs of the identical patients. 

### 3.1. Bone Mineral Densities and Mechanical Properties of the Samples

The BMDs of the cancellous bone samples averaged 315 ± 199 mg/cm² as measured by DXA and 347 ± 113 mg/cm³ measured by ashing. The mechanical properties of the bone samples were characterized by a structural modulus (*E*_*s*_) of 232 ± 151 N/mm² and ultimate compression strength (*σ*_max⁡_) of 6.4 ± 3.7 N/mm² (Table 1). The BMDs and mechanical parameters showed high standard deviations (Table 1).

### 3.2. Bone Mineral Densities of the Patients

The *T*-score of the patient's femoral neck averaged 0.31 ± 1.38 (range −1.8 to +2.6). This encompassed a broad distribution of bone densities (from normal to osteopenia bone). The results of the bone mineral densities of the patients presented as mean value ± standard deviation, minimum and maximum are given in Table 1. Therefore, patient DXA measurements of the femoral neck and head showed significant differences and high standard deviations, that is, ±166 and ±404 mg/cm², respectively (Table 1).

### 3.3. Correlation of Bone Mineral Densities with Mechanical Properties

A significant correlation exists between the DXA data on the bone samples derived from the centre of the femoral head and the DXA data from the patient measurements at the femoral head (*r* = 0.85, *P* < 0.01), whereas only a weak correlation was found between the DXA on the bone sample and the DXA on the patient measurements of the femoral neck, as shown in Table 2. 

Additionally, the best significant linear correlation was observed between ashing and DXA BMD on the bone samples (*r* = 0.92, *P* < 0.01). The structural modulus (*E*_*s*_) was also significant comparing DXA BMD femoral head (patient), bone sample, and ashing (*r* = 0.79, *r* = 0.82, and *r* = 0.8, *P* < 0.01) (Table 3). 

**Figure 2:**
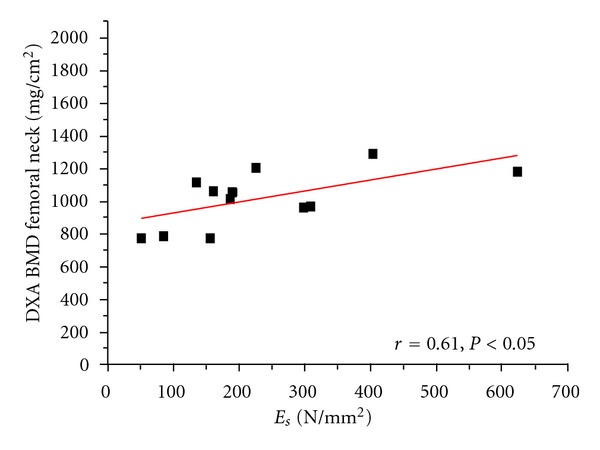
Linear correlation of structural modulus (*E*_*s*_) versus DXA BMD on femoral neck (standardised patient measurements).

**Figure 3:**
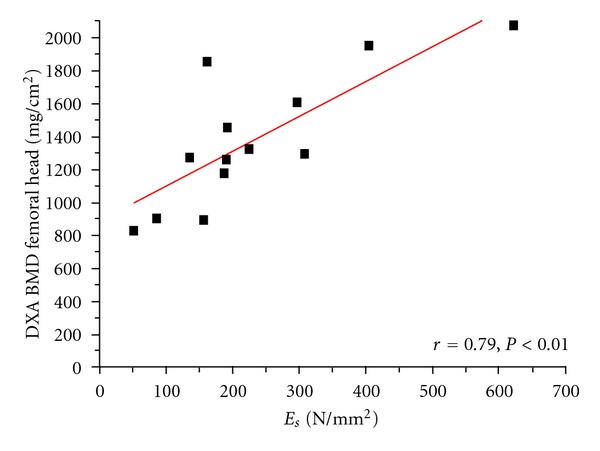
Linear correlation of structural modulus (*E*_*s*_) versus DXA BMD on femoral head (patient measurements).

**Figure 4:**
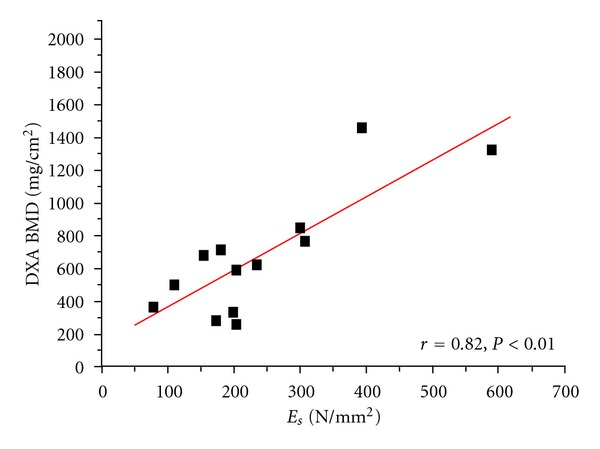
Linear correlation of structural modulus (*E*_*s*_) versus DXA BMD (on bone samples).

**Figure 5:**
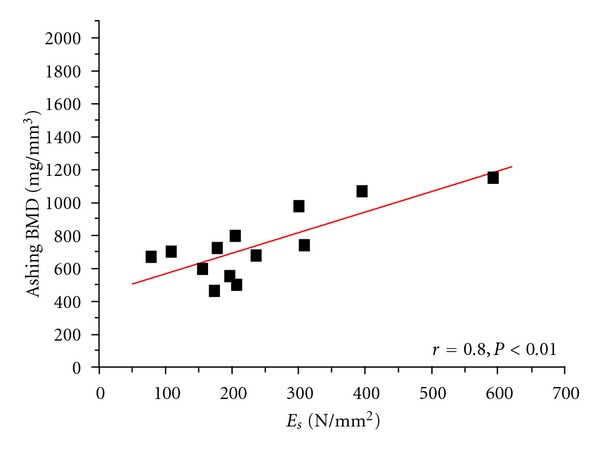
Linear correlation of structural modulus (*E*_*s*_) versus ashing BMD (on bone samples).

The distribution and linear correlations of the BMDs versus *E*_*s*_ are represented in Figures 2, 3, 4, and 5. Comparing the mechanical properties, DXA and ashing with the age of the patient revealed only low or no statistical correlations.  Table 4 illustrates the results of our measurements in comparison to other experimental studies. 

## 4. Discussion 

The aim of the present study is the comparison of mechanical properties and BMD measured at the femoral head and neck of osteoarthritic patients using ashing and DXA (*in vitro* and *in vivo*, resp.). For this, thirteen cancellous bone samples from human femoral heads retrieved from female patients with hip osteoarthritis were investigated. 

Bone samples of patients with osteoarthritis may be abnormal and show compositional changes, as investigated by Li and Aspden [17]. Therefore, we compared the mechanical and density properties of osteoarthritic bone with data on osteoporotic bone and bone samples from healthy donors (human femoral heads) post mortem [17]. Osteoarthritic bone samples revealed the greatest stiffness and apparent densities, and osteoporotic bone samples the lowest. Healthy and osteoporotic bone samples have shown similar linear regression relationships between mechanical and density properties [17]. 

Linear correlations have been observed between the mechanical properties and *in vitro* measured BMDs of bone samples, as well as the *in vivo* measured BMDs of the respective patients recorded by DXA. However, high standard deviations within the different measurements were found (Tables 1 to 3).

In different experimental studies, bone mineral density and material parameters of human cancellous femoral bone samples that retrieved post mortem were analysed by other research groups [10, 11, 18, 22]. Cody et al. [22] analysed male and female normal cancellous bone samples. They could demonstrate a DXA BMD at the femoral neck and greater trochanter of 650 ± 130 mg/cm² and 600 ± 150 mg/cm². In our present study, the patients' BMD amounted to 1016 ± 166 mg/cm² at the femoral neck and 1376 ± 404 mg/cm² at the femoral head. However, we only found BMD for the bone samples of about 315 ± 199 mg/cm². The reason for the lower BMD might be the measurement of osteoarthritic bone samples in contrast to other studies analysing samples derived from normal individuals [10, 22]. Uchiyama et al. [19] analysed bone samples derived from lumbar vertebrae and harvested at autopsy of pancreatic cancer fatalities. The comparison between the elastic modulus and BMD showed a correlation of *r* = 0.601, *P* < 0.005. Further *in vitro* examinations of cancellous femoral bone performed by Mazurkiewicz and Topoliński [21] showed good correlation between DXA BMD and ultimate compression strength of bone samples from patients with osteoporosis (*r* = 0.81) and hip osteoarthritis (*r* = 0.71).

The mechanical properties of bone samples are often described with the Young's modulus [9–11]. Sierpowska et al. [11] found a high average Young's modulus of 624.4 ± 213.9 N/mm² of cancellous bone (distal femur and proximal tibia) and an ultimate compression strength of 10.9 ± 4.2 N/mm². By contrast, Steinhauser et al. [10] determined values of 385.7 ± 189.4N/mm² for the Young's modulus and 8.5 ± 6.0 N/mm² for ultimate compression strength. Average values of 389 ± 270 N/mm², 7.4 ± 4.0N/mm² and 399 ± 130 mg/cm³ for Young's modulus, ultimate compression strength and mineral ash density of cancellous bone from healthy human femoral heads post mortem, respectively, were obtained by Rohlmann et al. [18]. Both the mechanical parameters and BMD of ashing represent higher values compared to our results (Table 4) and could depend on the fact that the investigation was done on healthy instead of osteoarthritic bone material as was used in our study. Our samples were prepared in a single orientation, so that the distribution of the Young's modulus was not due to anisotropy [4]. Bone is a complex material made of different organic and inorganic components; hence, the structural modulus (*E*_*s*_) describes the Young's modulus (mechanical properties) more accurately in conjunction with bone samples [23].

However, the correlation between properties as well as bone mineral density (Table 3) of retrieved bone samples and the bone density of the femoral head and neck previously measured in the same patients has not been investigated so far. Measurements of patient bone mineral density by DXA is a noninvasive technique and beneficial for determining fracture risk [20]. Au et al. [20] measured an average BMD of 758 ± 114 mg/cm² in the femoral neck of healthy female patients. In our study, a higher patient BMD of the neck (1016 ± 166 mg/cm²) in osteoarthritic patients was found, as theoretically expected (Table 4). This may depend in part on the location of patients' DXA measurements at the femoral neck, in contrast to the *in vitro* measurements of the bone samples using the femoral head. This could also explain the low correlation coefficient between each patient's DXA BMD of the femoral neck and the mineral density of the bone sample as determined *in vitro* by DXA and ashing. On the other hand, each patient's DXA BMD of the femoral head demonstrated a significant linear correlation (Table 2). Therefore, a specific orientation for *in vitro* BMD measurements of bone samples is recommended in order to enable comparison to BMD calculations accomplished *in vivo*. In further studies, the presented results of osteoarthritic bone should be compared with bone samples from healthy individuals and osteoporotic patients of the same age.

In summary, a strong relationship was found between the mechanical properties and BMDs of retrieved cancellous samples from the femoral head and the BMDs of the respective patients measured *in vivo*. However, adequate orientation of both *in vitro* and *in vivo* DXA measurements at the proximal femur is required. The linear correlations found between mechanical data and bone mineral densities can help to determine the mechanical load capacity of individual patients in terms of surgical treatments by means of non-invasive bone density measurements preoperatively. In turn, this knowledge of mechanical properties could be useful for patient treatment, for example, in total joint arthroplasty, by choosing an adequate endoprosthetic implant adapted to the quality of the individual bone stock. Additional DXA measurements of the femoral head could derive useful data on the BMD for adequate fixation of implants like dynamic hip screws within the femoral head.

## Figures and Tables

**Table 1:** Mean value ± standard deviation (SD), minimum and maximum of bone mineral densities and mechanical properties of 13 bone samples.

	DXA BMD femoral neck	DXA BMD femoral head	DXA BMD	Ashing BMD	*E*_*s*_	*σ*_max⁡_
	[mg/cm²]	[mg/cm²]	[mg/cm²]	[mg/cm³]	[N/mm²]	[N/mm²]
	Patient measurements	Patient measurements	Bone samples	Bone samples	Bone samples	Bone samples
Mean ± SD	1016 ± 166	1376 ± 404	315 ± 199	347 ± 113	232 ± 151	6.4 ± 3.7
Min	768	823	97	199	51	1.9
Max	1289	2081	734	567	624	13.5

**Table 2:** Linear correlation between the BMD measured on each patient and BMD of the respective bone samples.

	DXA BMD femoral neck	DXA BMD femoral head	DXA BMD	Ashing BMD
	Patient measurements	Patient measurements	Bone samples	Bone samples
DXA BMD femoral neck	1	0.78**	0.67*	0.49
DXA BMD femoral head	0.78**	1	0.85**	0.79**
DXA BMD	0.67*	0.85**	1	0.92**
Ashing BMD	0.49	0.79**	0.92**	1

*Correlation is significant at the 0.05 level (2-tailed).

**Correlation is significant at the 0.01 level (2-tailed).

**Table 3:** Linear correlation between BMD (DXA and ashing) and mechanical properties (structural modulus (*E*_*s*_) and ultimate compression strength (*σ*_max⁡_)).

	DXA BMD femoral neck	DXA BMD femoral head	DXA BMD	Ashing BMD
	Patient measurements	Patient measurements	Bone samples	Bone samples
*E*_*s*_	0.61*	0.79**	0.82**	0.8**
*σ*_max⁡_	0.36	0.56*	0.61*	0.67*

*Correlation is significant at the 0.05 level (2-tailed).

**Correlation is significant at the 0.01 level (2-tailed).

**Table 4:** Results of BMD testing and mechanical properties of bone samples from the literature compared to own test data.

	DXA BMD femoral neck	DXA BMD femoral head	DXA BMD	Ashing BMD	*E*_*s*_	*σ*_max⁡_
	[mg/cm²]	[mg/cm²]	[mg/cm²]	[mg/cm³]	[N/mm²]	[N/mm²]
	Patient measurements	Patient measurements	Bone samples	Bone samples	Bone samples	Bone samples
Rohlmann et al., 1980 [18]				399 ± 130	389 ± 270	7.4 ± 4.0
Uchiyama et al., 1999 [19]			88 ± 16		126 ± 96.9	
Au et al., 1998 [20]	758 ± 114					
Mazurkiewicz and Topoliński, 2009 [21]			289 ± 69			13.4 ± 6.5
Own data	1016 ± 166	1376 ± 404	315 ± 199	347 ± 113	232 ± 151	6.4 ± 3.7
